# Recent advances in the development of portable technologies and commercial products to detect Δ^9^-tetrahydrocannabinol in biofluids: a systematic review

**DOI:** 10.1186/s42238-024-00216-0

**Published:** 2024-02-27

**Authors:** Pierrick Clément, Walter K. Schlage, Julia Hoeng

**Affiliations:** 1 Centre Suisse d’Electronique Et de Microtechnique SA (CSEM), Rue Jaquet-Droz 1, 2002 Neuchâtel, Switzerland; 2 Biology Consultant, Max-Baermann-Strasse 21, 51429 Bergisch Gladbach, Germany; 3 Vectura Fertin Pharma, C/O Jagotec AG, Messeplatz 10, 4058 Basel, Switzerland

**Keywords:** Δ^9^-tetrahydrocannabinol, THC detection, Biosensor, Biofluids, Cannabis, Phytocannabinoids

## Abstract

**Background:**

The primary components driving the current commercial fascination with cannabis products are phytocannabinoids, a diverse group of over 100 lipophilic secondary metabolites derived from the cannabis plant. Although numerous phytocannabinoids exhibit pharmacological effects, the foremost attention has been directed towards Δ^9^-tetrahydrocannabinol (THC) and cannabidiol, the two most abundant phytocannabinoids, for their potential human applications. Despite their structural similarity, THC and cannabidiol diverge in terms of their psychotropic effects, with THC inducing notable psychological alterations. There is a clear need for accurate and rapid THC measurement methods that offer dependable, readily accessible, and cost-effective analytical information. This review presents a comprehensive view of the present state of alternative technologies that could potentially facilitate the creation of portable devices suitable for on-site usage or as personal monitors, enabling non-intrusive THC measurements.

**Method:**

A literature survey from 2017 to 2023 on the development of portable technologies and commercial products to detect THC in biofluids was performed using electronic databases such as PubMed, Scopus, and Google Scholar. A systematic review of available literature was conducted using Preferred Reporting Items for Systematic.

Reviews and Meta-analysis (PRISMA) guidelines.

**Results:**

Eighty-nine studies met the selection criteria. Fifty-seven peer-reviewed studies were related to the detection of THC by conventional separation techniques used in analytical laboratories that are still considered the gold standard. Studies using optical (*n* = 12) and electrochemical (*n* = 13) portable sensors and biosensors were also identified as well as commercially available devices (*n* = 7).

**Discussion:**

The landscape of THC detection technology is predominantly shaped by immunoassay tests, owing to their established reliability. However, these methods have distinct drawbacks, particularly for quantitative analysis. Electrochemical sensing technology holds great potential to overcome the challenges of quantification and present a multitude of advantages, encompassing the possibility of miniaturization and diverse modifications to amplify sensitivity and selectivity. Nevertheless, these sensors have considerable limitations, including non-specific interactions and the potential interference of compounds and substances existing in biofluids.

**Conclusion:**

The foremost challenge in THC detection involves creating electrochemical sensors that are both stable and long-lasting while exhibiting exceptional selectivity, minimal non-specific interactions, and decreased susceptibility to matrix interferences. These aspects need to be resolved before these sensors can be successfully introduced to the market.

**Supplementary Information:**

The online version contains supplementary material available at 10.1186/s42238-024-00216-0.

## Background

*Cannabis sativa* (commonly called cannabis) is an ancient plant with multiple usages in many cultures, including its application as textile fiber and edible oil, and for its pharmacological activities (Radwan et al. 2021). Owing to its psychoactive effects, cannabis, and cannabis resin are both listed in Schedules I and IV of the United Nations 1961 Single Convention on Narcotic Drugs (Gonçalves et al. 2019). Since the legalization of cannabis use for medical, dietary, and recreational purposes under various legislations around the world, the market for cannabis products has grown considerably (Kitdumrongthum and Trachootham 2023; Donnan et al. 2023; Gonçalves et al. 2019). The major compounds responsible for the recent commercial interest in cannabis products are cannabinoids, a class of more than 100 different lipophilic secondary metabolites of the cannabis plant that are mainly produced and stored in the inflorescences (i.e., the part of the plant that bears blossoms) of female cannabis plants (Gonçalves et al. 2019). Although many phytocannabinoids have been found to possess pharmacological activity, the two most abundant phytocannabinoids, Δ9-tetrahydrocannabinol (THC) and cannabidiol (CBD) have garnered the greatest interest for human use to date. Although they are structurally closely related, THC and CBD differ in their psychotropic activity; THC causes significant psychological effects, whereas CBD does not (Singh et al. 2022; Gonçalves et al. 2019).

By specific breeding, cannabis varieties can be modified to express high levels of THC and low levels of CBD, or vice versa. The latter varieties are also known as hemp and their cultivation and use are now legal in many countries. By contrast, THC-rich cannabis, also termed marijuana, and THC-containing products are strictly regulated or even forbidden as a narcotic in many jurisdictions (Gonçalves et al. 2019). The legal threshold level for THC in hemp products or in cannabis extracts varies in different countries; for example, the threshold is 0.3% in the United States (US) and the European Union and 1% in Switzerland.

A critical issue for consumers is the amount of THC in blood plasma after cannabis consumption. In an investigation of traffic accidents in the United Kingdom (UK) in 2014, THC blood levels ≥ 5 µg/L were associated with a significant increase in crash risk, whether or not ingestion had occurred recently, and regardless of medicinal or illicit drug use origin (Wolff and Johnston 2014). It remains a matter of debate whether the blood cut-off level of 5 µg/L within the so-called ‘impairment window’ after cannabis use is an adequate indicator of driving impairment, or whether more sophisticated testing is needed (Di Ciano et al. 2023; DeGregorio et al. 2021; Wennberg et al. 2023; Preuss et al. 2021).

Numerous factors have been identified that strongly influence the pharmacokinetics of THC in individuals and the relationship between THC levels in various samples (e.g., blood, urine, saliva, and exhaled breath) and driving impairment (DeGregorio et al. 2021; Wurz and DeGregorio 2022). As such, there appears to be a lack of universally applicable per se limits for these types of bodily fluids.

Irrespective of the uncertainties concerning correct sampling and testing strategies, adequate sample matrices (including saliva and sweat), and meaningful cut-off values, there is an obvious demand for sensitive and fast measurement of THC (and its metabolites and accompanying phytocannabinoids). Furthermore, there is a need for a method of THC measurement that can provide reliable, immediate, and affordable analytical data comparable to those obtained offline using combined chromatographic and mass spectrophotometric methods, which currently represent the gold standard but require extensive laboratory infrastructure. This review aims to provide an overview of the current status of such alternative technologies enabling non-invasive THC measurements through the use of on-site mobile devices or personal monitoring. The need for immediate monitoring of phytocannabinoids is growing, for example, in personalized medicine applications for exact dose control. THC was chosen as the subject of focus because the legal situation has generated a high demand for THC measurements.

## Methods

A literature search was conducted of peer-reviewed articles written in English and published from January 2017 to August 2023 using PubMed, Scopus, and Google Scholar databases. The objective was to screen for innovative sensing technologies for the detection of THC, based on searches for analytes, biofluids, and types of sensors. The following keywords were selected for each parameter:

- Analyte: THC detection, tetrahydrocannabinol detection, cannabinoid detection
- Biofluids: saliva, oral fluid, blood, sweat, breath, urine
- Types of sensors: electrochemical sensor, optical sensor, bioassay, biosensor, field-effect transistor, aptasensor

More details on the search methodology can be found in Additional file 1 and the PRISMA flow chart in Additional file 2.

A total of 8893 publications were identified and further filtered to exclude studies that did not report on the detection/quantification of THC, media that were not biofluids, synthetic cannabinoids, post-mortem analyses, other less commonly available body fluids (e.g., breast milk and semen), and duplicates (from the different combinations of search terms). This process resulted in narrowing down the initial search to 184 manuscripts. Each article was reviewed blindly to gather the following information: detection technique, sensing layer composition, concentration range, biofluids tested, selectivity, limit of detection (LOD), detection time, reusability, and robustness (i.e., pH, biofouling, temperature, and chemicals). Finally, 95 articles were discarded as they were deemed to be outside the scope of this review, resulting in 89 papers being included in this study.

## THC sensing technologies

### THC sensing by conventional analytical techniques

In total, 57 articles, representing 64% of the publications included in this review, were related to the detection of THC by conventional separation techniques that are used in analytical laboratories, such as liquid chromatography (LC), ultra-high-performance LC, and gas chromatography. This indicates that these techniques are currently the most widely used for the detection, identification, separation, and quantification of THC. These methods are often coupled with mass spectral detection methods (e.g., mass spectroscopy [MS], and tandem MS–MS).

Chromatographic methods serve as tools for separating compound mixtures extracted from any source. The process involves dissolving the mixture of molecules (from the sample) and introducing them into an instrument, where they are passed through a separation column. This column is either filled or coated with a stationary phase that exhibits varying affinity towards the different compounds based on their chemical properties. By utilizing different mobile phases, the rate at which the molecules progress and traverse the column determines the elution time of each compound, resulting in the separated compounds reaching the detector at distinct time points. The latest advancements in chromatographic techniques fall beyond the coverage of this review so we have chosen only articles from 2020 onwards for comparison with alternative technologies further described. Table 1 summarises the techniques encountered.

**Table 1:** Conventional analytical techniques for THC sensing in biofluids

Technique	Coupling	Media	Limit of detection (ng/mL)	Reference
Liquid chromatography	High-resolution MS	Blood, breath	1	(Wurz and DeGregorio 2022)
		Plasma	0.2	(Mohamed et al. 2021)
		Blood	0,4	(Joye et al. 2020)
		Blood	0.5	(DeGregorio et al. 2020)
Liquid chromatography	MS/MS	Plasma	0.78	(Sempio et al. 2022)
		Blood, urine	1	(Reber et al. 2022)
		Serum	0.2	(Scheunemann et al. 2021)
		Urine	1	(Reber et al. 2021)
		Dry urine spot	2	(Moretti et al. 2021)
		Blood	2	(Klu et al. 2021)
		Dry oral spot	2	(Gorziza et al. 2021)
		Urine	0.6	(Goggin and Janis 2021)
		Oral fluid	1	(Coulter and Wagner 2021)
		Blood, breath	0.5	(Hubbard et al. 2020)
		Plasma	0.5	(da Silva et al. 2020)
		Oral fluid	1	(Bassotti et al. 2020)
Ultra high-performance liquid chromatography	MS/MS	Plasma	0.87	(Manca et al. 2022)
		Oral fluid	0.1	(Lin et al. 2022)
		Plasma	0.2	(Ahmed et al. 2022)
		Blood, oral fluid, sweat	0.2 (blood, oral fluid), 0.5 (sweat)	(Pichini et al. 2020)
Gas chromatography	MS/MS	Blood, urine	0.15	(Frei et al. 2022)
Paper spray	MS/MS	Saliva	0.78	(Borden et al. 2022)

*MS* Mass spectroscopy

The LODs for the chromatographic techniques are mostly below the ng/mL range, which is lower than THC legal cut-off values. Furthermore, these studies have demonstrated excellent selectivity for THC among THC metabolites.

Although gas chromatography and LC-based techniques provide precise, consistent, and specific detection of cannabinoids, their intricate setup constraints prevent their use for on-site detection of THC as these instruments lack portability. Furthermore, prior to being injected and analyzed by any chromatography-based method, the samples must undergo extraction and additional pre-treatment procedures. These pre-treatment processes are laborious, demand expertise, and can potentially introduce errors in the identification of THC.

An alternative method using paper spray MS was also identified. The coupling of reactive paper spray with MS allowed for the direct, quantitative analysis of THC without additional sample handling.

Overall, the development of portable instruments, such as biosensor platforms, is necessary to facilitate swift screening and identification processes, notably for on-site testing. This would eliminate the need to send samples to a laboratory for analysis, which is particularly crucial in situations where immediate results are required, such as law enforcement settings, workplace drug testing, or roadside testing for impaired driving. Portable sensors should offer real-time detection, allowing for quick decision-making and appropriate action.

### Sensors for rapid on-site THC detection

**Fig. 1:**
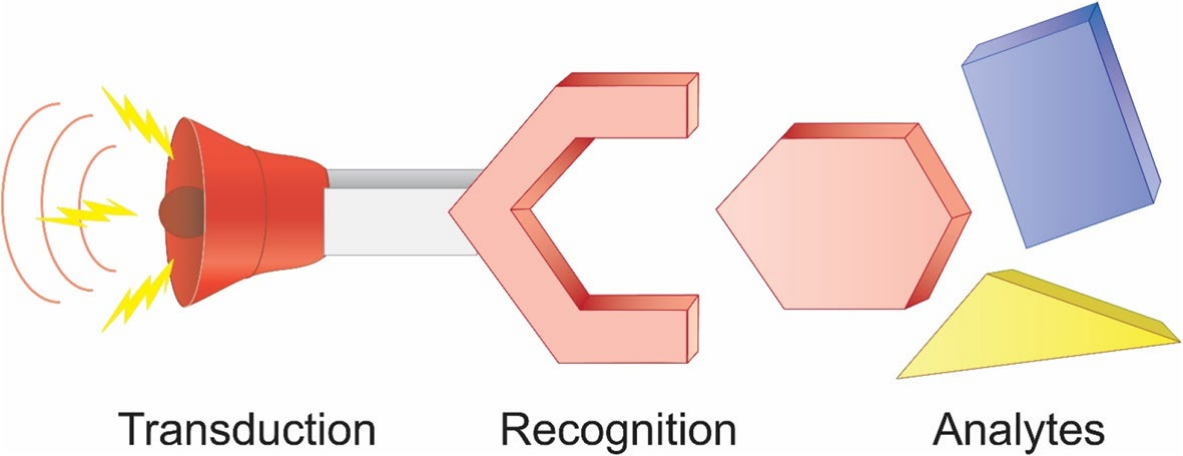
Scheme representing a biosensor working principle. The target analyte should specifically interact with the recognition element which event is signaled by the transduction element

The utilization of portable sensors is a potential alternative approach for THC detection. Sensing platforms have been extensively applied in diagnostics, biomedicine, food safety, environmental monitoring, defense, and security. A sensor is characterized as an integrated device that enables the quantitative or semi-quantitative measurement of signals. A standard sensor is composed of three components: the recognition element, interface, and transducer (Fig. 1). For biosensors, the recognition element can be nucleic acids, antibodies, enzymes, or whole cells, enabling the detection of the target molecule (THC in the context of this review).

The immobilization of the recognition element is at the interface with the transducer and, subsequently, the transducer converts the molecular recognition event into a detectable signal. The transducer can originate from various sources, including optical (e.g., absorbance, luminescence, or fluorescence), electrochemical (e.g., potentiometric or amperometric), or acoustic (e.g., quartz crystal microbalance, surface acoustic wave, or surface transverse wave) mechanisms. Some portable biosensing platforms have been described in recent review articles for the on-site analysis of illicit drugs (Harpaz et al. 2022; Purohit et al. 2020; Klimuntowski et al. 2020; Anzar et al. 2022; Amini et al. 2022; Ahmed et al. 2020).

### Optical sensors

Optical sensing methods identified in the publications included in this review are divided into three categories: surface-enhanced Raman spectroscopy (SERS); ultraviolet–visible-near infrared spectroscopy; and colorimetric, fluorescence, and chemiluminescence (Table 2).

**Table 2:** Optical sensing methods for the detection of THC

Type	Mechanism	Medium	LOD	Cross-reactivity tests	Reference
SERS	Diatomaceous earth-Au NPs thin-layer chromatography in tandem with surface-enhanced Raman spectroscopy (TLC-MS)	Saliva, urine	N.A	None	(Sivashanmugan et al. 2019a, b, c)
	Ag NPs supported diatom frustules as SERS active substrate	Plasma, saliva	0.31 ng/mL	THC-COOH, THC-OH	(Sivashanmugan et al. 2019a, b, c)
	Ag NPs SERS-active dendritic structure with PCA	Saliva	N.A	Cocaine, heroin, oxycodone,	(Dies et al. 2018)
UV–vis-NIR-spectroscopy	Portable MicroNIR spectrometer	Saliva	10 ng/mL	None	(Risoluti et al. 2019)
	Paper-based lateral flow competitive immunoassay with anti-THC-Au NPs, sensing via photothermal radiometry	Saliva	5 ng/mL	None	(Hayden et al. 2022)
	Lateral flow immunoassays with lock-in thermography	Saliva	2 ng/mL	None	(Thapa et al. 2020)
Colorimetric and chemiluminescence	Membrane with captured THC antibody-AuNPs, competitive immunoassay	Saliva	0.17 ng/mL	None	(Yu et al. 2021)
	Competitive immunoassay with THC antibody conjugated fluorescent nanoparticles	Saliva	1 pg/mL	CBD	(Liang et al. 2022)
	Capillary-based competitive enzyme-linked immunosorbent assay	Sweat	0.02 ng/mL	Methadone, METH, amphetamine	(Xue et al. 2020)
	Fluorescent-based immunoassay with THC antibody conjugated fluorescent nanoparticles	Saliva	0.01 ng/mL	None	(Plouffe and Murthy 2017)
	Upconverting nanoparticle-based lateral-flow immunoassay with immunoglobulin G and streptavidin	Saliva	2 ng/mL	None	(Chand et al. 2021)

Spectroscopy relies on the interaction of matter with light and other forms of radiation, where absorption and emission play crucial roles. This method frequently entails the division of light (electromagnetic radiation) into its individual wavelengths, forming a spectrum. Recently, SERS sensing technology demonstrated that THC detection was possible in biofluids. Sivashanmugan et al. reported the use of electroless deposition of Ag nanoparticles (NPs) coupled on diatom biosilica surfaces to form hybrid photonic-plasmonic modes, which can generate a strong local electromagnetic field and enhance SERS signals (Sivashanmugan et al. 2019a, b, c). The principal component analysis method was applied to differentiate SERS spectra of THC (1 pM) in methanol, plasma, and purified saliva samples.

Following the same principle, Dies et al*.* further developed a thin-layer chromatography-SERS with AuNPs integrated on diatom biosilica surfaces. The thin-layer chromatography method was required to eliminate the influence of urea in urine samples. Another study used the electric field-guided assembly of AgNPs in a dendritic fashion on a silicon substrate, allowing high sensitivity for the detection of THC and other illicit drugs in saliva samples (Dies et al. 2018). Risoluti et al. (2019) developed a miniaturized and portable analytical platform based on micro near-infrared spectrometry associated with chemometric analysis to simultaneously detect and quantify traces of THC in oral fluids without sample pre-treatment. Using a partial least square-discriminant analysis model of prediction, they were able to detect THC concentrations from 10 to 100 ng/mL, with a precision and a sensitivity of about 1.51% and 0.1%, respectively.

**Fig. 2:**
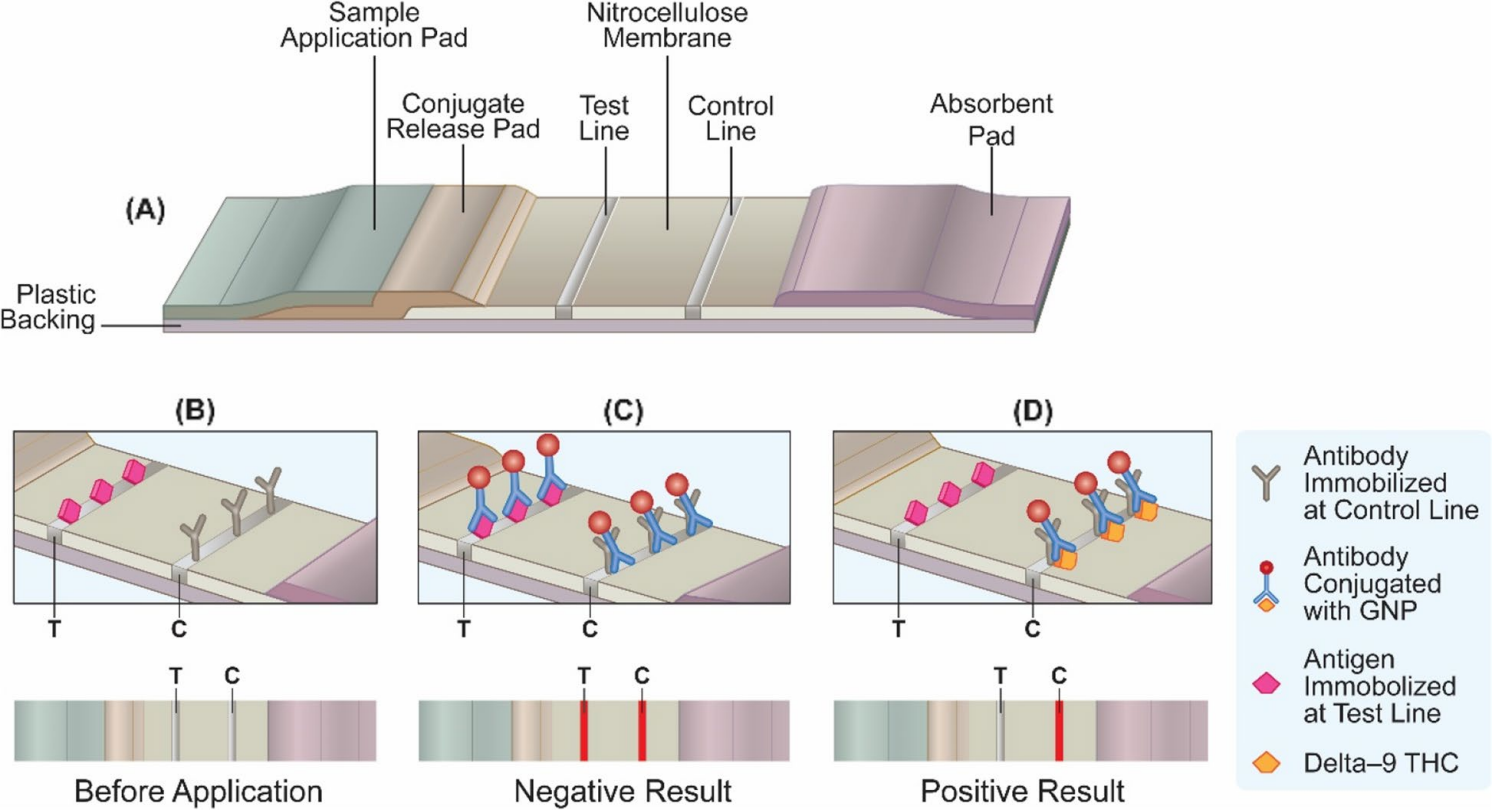
**a** Schematic diagram of LFIA test strip. Schematic illustration of one binding mechanism of competitive LFIAs, **b** before use, **c** negative test, and **d** positive test with their respective visual appearance

Many articles employed the immunoassay principle, which is a widely used method in medical diagnostics and research to detect and measure the presence and concentration of specific substances in biological samples. Immunoassays rely on the specific binding between an antigen (here, THC) and an antibody (a protein that recognizes and binds to the antigen). Antibodies are produced by the immune system in response to the presence of foreign substances (antigens) in the body and each antibody is highly specific to its corresponding antigen. Lateral flow immunoassays (LFIAs) provide quick and qualitative results without the need for complex laboratory equipment (Fig. 2). They operate on the following principles:

1. Sample pad: a sample, such as blood, urine, or saliva, is applied to a sample pad. This pad serves as the entry point for the sample and allows it to flow along the cellulose test strip.
2. Conjugate pad: the sample flows from the sample pad to a conjugate pad containing conjugated particles. These particles are typically AuNPs or colored latex beads that are conjugated with antibodies or antigens specific to the target analyte.
3. Test line(s): the test strip contains one or more test lines, typically immobilized antibodies or antigens, that are specific to the target analyte. These lines are positioned in a way such that the flow of the sample and conjugate particles will pass through them.
4. Control line: in addition to the test line(s), a control line is also present on the strip. The control line contains a capture molecule, such as an antibody, that captures the excess conjugate particles, serving as validation that the test is working properly.
5. Flow: as the sample and conjugate particles flow along the strip, they encounter the test line(s) and control line. If the target analyte is present in the sample, it will bind to the conjugate particles, forming a complex.
6. Detection: the complex formed between the target analyte, conjugate particles, and the corresponding antibodies or antigens in the test line(s) will accumulate at the respective line(s), resulting in a visible signal. The control line will always show a signal if the completed test is functioning correctly.
7. Interpretation: the presence or absence of the signal in the test line(s) indicates the presence or absence of the target analyte in the sample. The intensity of the signal correlates with the concentration of the analyte.

Hayden et al. (2022) developed a platform based on Arduino that utilizes low-cost far infrared, single-element detectors to offer sensitive and semi-quantitative results from commercially available LFIA-based rapid tests (NarcoCheck®, Kappa City Biotech, Montluçon, France). This uses a competitive immunoassay with THC antibodies conjugated to AuNPs, which act as visible indicators in both the test and control lines. The target analyte blocks the binding sites in the test line, allowing the antibodies to pass the test line and instead bind to the control line. Using this method, they were able to improve the THC detection limit in the saliva of the rapid test from 25 to 5 ng/mL. Similarly, Thapa et al. developed a thermo-photonic imaging system that utilizes commercially available low-cost LFIAs (Thapa et al. 2020). Their reader technology examined photothermal responses of AuNPs in LFIA through lock-in thermography. It allowed them to improve the commercial LFIA limit of detection (25 ng/mL) to as low as 2 ng/mL in saliva with an accuracy of 96%.

**Fig. 3:**
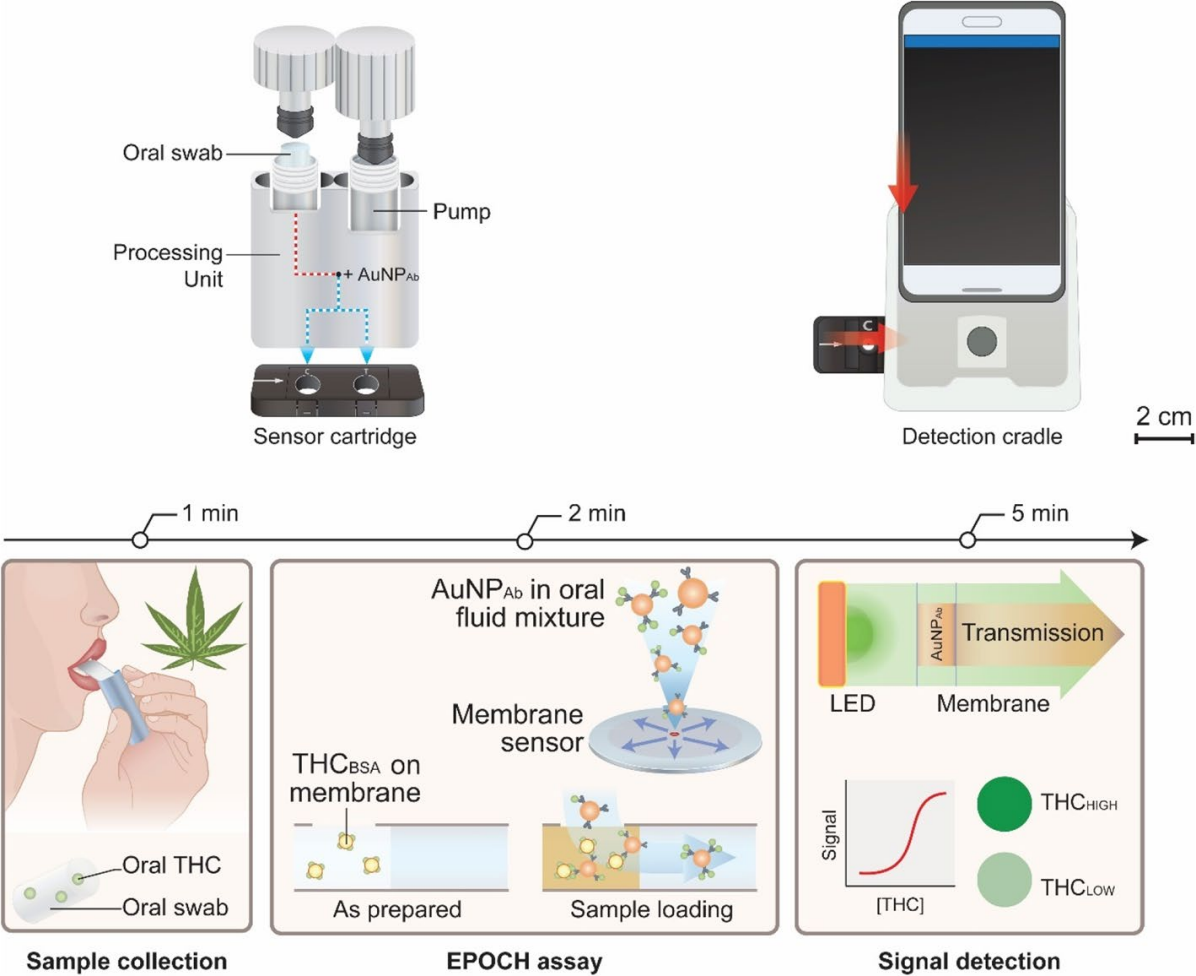
The EPOCH system has three modules for on-site THC assay: (i) a sample processing kit for extracting oral fluid and labeling with AuNP-THC antibody, AuNP_ab_; (ii) an injection-molded cartridge housing membrane sensors; and (iii) a detection cradle for optical signal detection. The processing kit, paired with the sensor cartridge, delivers AuNPAb-oral fluid mixture to test and control sites. The sample-spotted cartridge is inserted into the cradle and imaged by a smartphone camera. 5-min THC detection. (Left) An oral-fluid sample is collected using a swab. (Middle) oral fluid is extracted and mixed with AuNP_ab_. The mixture is then spotted on a radial membrane sensor that has immobilized THC competitors (THC haptens conjugated to bovine serum albumin carriers; THCBSA). (Right) AuNP_ab_ differentially binds to THCBSA according to oral THC concentration. Transmission through the sensing spot is digitized for THC quantification

Yu et al. developed a particularly interesting approach for the detection of THC in saliva using an express probe for on-site cannabis inhalation (Fig. 3) (Yu et al. 2021). This compact test module comprised an oral fluid processing kit, a sensor cartridge, and an optical detection unit. The saliva is mixed with THC antibody-conjugated AuNPs and spotted on a radial membrane sensor cartridge with immobilized THC competitors. A 525-nm light-emitting diode was used to measure the optical signal of AuNPs by transmission through the sensing spot with a smartphone camera. They achieved an LOD of 0.17 ng/mL and the test was unaffected by consumption of coffee, alcohol, or tobacco. Following this principle, Liang et al. developed a competitive immunoassay paper microfluidic chip with anti-THC-conjugated fluorescent NPs (Liang et al. 2022).

The read-out was performed using a smartphone-based fluorescent microscope for counting in the test zone. They were able to detect THC in saliva with a LOD of 1 pg/mL without cross-reactivity to CBD. The quantification of THC was performed with machine learning techniques to eliminate sample-to-sample variation. Through classification algorithms, they showed 88% accuracy for six different concentrations of THC. Furthermore, independent validation of the sample set was performed, identifying positive samples at 100% accuracy and quantifications at 80% accuracy.

Xue et al. developed a system based on capillary arrays combined with competitive enzyme-linked immunosorbent assay (ELISA) to detect THC spiked in sweat (Xue et al. 2020). The inner surface of each capillary was first coated with a THC antibody and then the mixed solution containing free THC and a THC horseradish peroxidase (HRP) conjugate competed for the limited amount of antibody for 15 min. The sample was further ejected from the capillary and rinsed with buffer. The chemiluminescent substrate, which is oxidized in the presence of HRP, was injected and chemiluminescent images were recorded using a complementary metal-oxide semiconductor camera after 3 s of incubation. The dynamic range of concentrations could be tuned by changing the concentration of THC-HRP (competitor). Following this principle, they were able to detect methadone, methamphetamine, amphetamine, and THC with LODs of 1.6, 142, 35, and 20 pg/mL, respectively.

Plouffe et al. improved standard colorimetric LFIA (THC LOD of 5 ng/mL) by replacing AuNPs with polymeric fluorescent NPs to conjugate THC antibodies, which are more sensitive and allowed quantification (Plouffe and Murthy 2017). A fluorescent microscope was used to visualize the presence of the analyte with an excitation wavelength of 480 ± 30 nm coupled to a red-filtered (605 ± 50 nm) charge-coupled device camera. With this method, a LOD of > 0.1 ng/mL of THC spiked in saliva samples was successfully achieved with a linear detection range even at concentrations < 5 ng/mL. Three double-blind tests were performed, and it was determined that the LFIA quantified the concentration within 10% of the actual value, based on the average measured values. However, variations remain in the measured values with 14–40% variability.

Another approach was developed by Chand et al. with an upconverting NP (UCNP)-based LFIA (Chand et al. 2021). UCNPs convert near-infrared excitation into visible emissions. Contrary to standard LFIA biosensors, the researchers integrated an additional enhancement pad between the conjugate pad and nitrocellulose membrane. UCNPs dually conjugated with THC-specific immunoglobulin G (IgG) and streptavidin (SA) and UCNPs conjugated with biotin (UCNP–biotin) were dried in the conjugated pad and enhanced pad, respectively. UCNP–IgG–SA, upon interacting with THC, flow through the enhanced pad and bind with UCNP–biotin, consequently forming bright UCNP clusters on the test and control zones. The test signals were captured after an assay time of 20 min. An experimental THC LOD of 2 ng/mL in saliva was achieved with a linear detection range of 2–15 ng/mL.

### Electrochemical sensors

**Fig. 4:**
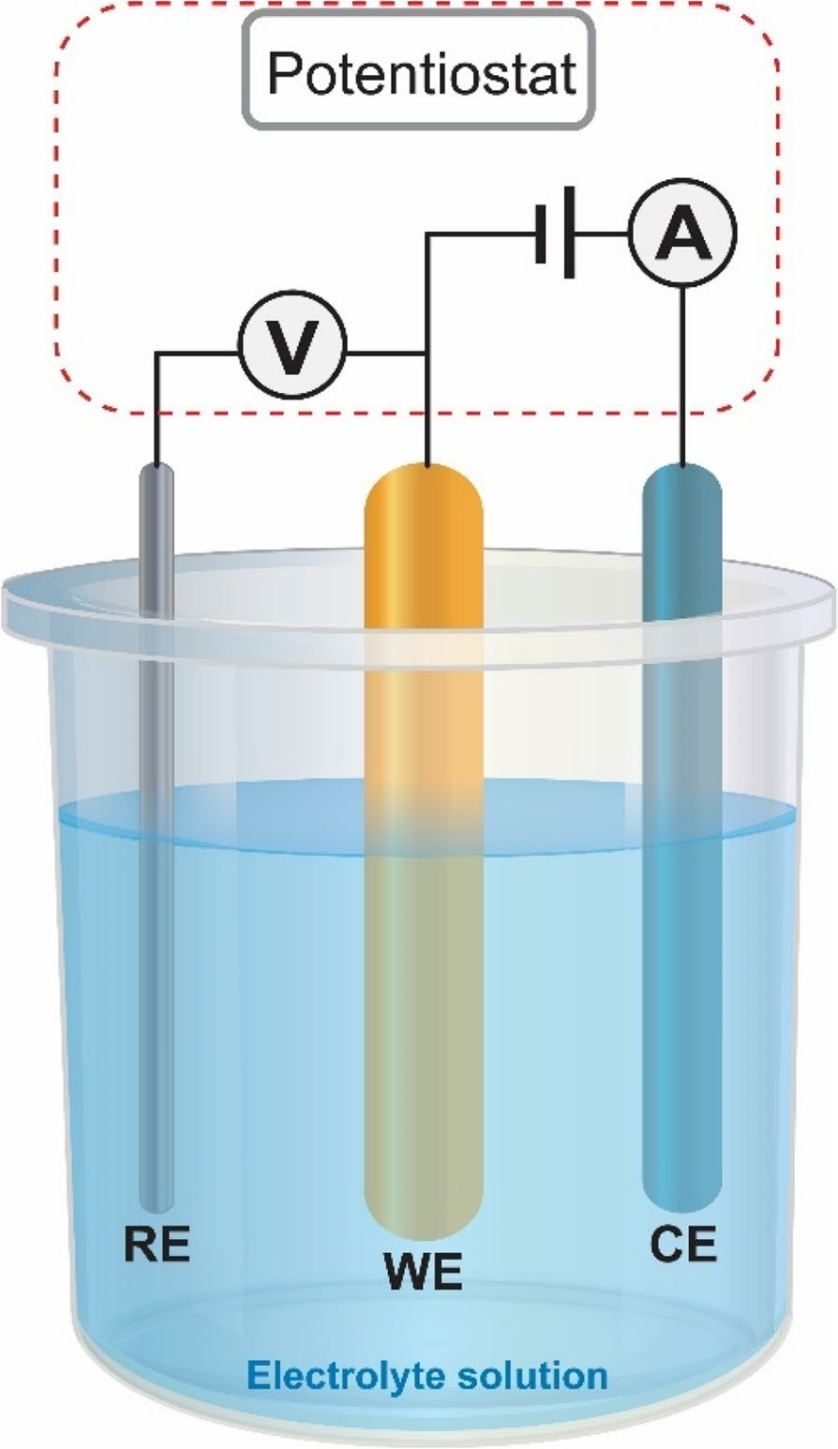
*Electrochemical biosensors with three electrodes: reference (RE), working* (WE), and counter (CE) connected to a potentiostat

The field of electrochemical sensors has witnessed significant advancements in various domains, including clinical diagnosis, quality control in food processing, and environmental monitoring. Notably, the incorporation of nanotechnology in electrochemical sensors has led to the development of nano-inspired sensors capable of monitoring molecular interactions at the nanoscale. This has enabled the realization of reagent-free, label-free, non-invasive, on-site, and in-situ measurements of parameters of interest in diverse matrices or media. Electrochemical sensors are based on measuring chemical event-dependent changes in the conductance, resistance, or capacitance of the sensor (Fig. 4). To perform these measurements, three kinds of electrodes are generally used:

- The reference electrode provides a constant and defined potential and is used to normalize the working electrode potential. The most widely employed is the Ag/AgCl and the saturated calomel electrode (Hg/HgCl_2_).
- Working electrode, which monitors the oxidation or the reduction of a species near the surface of the electrode. The working electrode is typically made from a chemically stable and conductive material such as gold, platinum, or glassy carbon. Its surface morphology and functionalization are key parameters as they define the selectivity, sensitivity, and robustness of the biosensor.
- Counter electrode, which completes the circuit and allows charges to flow and measure (or apply) a current. These are fabricated from inert and conductive materials.

Electrochemical sensors employ different types of electrochemical detection techniques, such as amperometry (measurable current), potentiometry (measurable potential), impedance spectroscopy (measurable resistance and capacitance equivalent elements), and field-effect transistors, that measure current as a result of a potentiometric effect at a gate electrode. Details on the most common measurement techniques, including differential pulsed voltammetry (DPV), square wave voltammetry (SWV), cyclic voltammetry, and electrochemical impedance spectroscopy, can be found elsewhere (Grieshaber et al. 2008). The electrochemical sensor technologies identified in this review are summarised in Table 3.

**Table 3:** Electrochemical sensing technology for the detection of THC

	Mechanism	Medium	Limit of detection	Cross-reactivity tests	Reference
DPV	THC electrochemical oxidation on SPCEs	Artificial saliva diluted to 50% with buffer	42 ng/mL	pH	(Williams et al. 2023)
	THC electrochemical oxidation on CuPc/SPGE	Saliva diluted with 20:80% of ethanol:buffer	1.4 ng/mL	SCN, pH, CH_3_COO^−^, NO_3_^−^, Cl^−^, H_2_PO_4_^−^, SO_3_^2−^, F^−^, CO_3_^2−^, NO_2_^−^, I^−^, SO_4_^2−^, K^+^, Na^+^, NH_4_^+^, Mg^2+^, citric acid, glucose, cysteine, and ascorbic acid	(Pholsiri et al. 2023)
	Competitive binding between THC and redox indicator with an aptamer on Au electrode	Buffer and saliva	1.6 ng/mL	None	(Kekedy-Nagy et al. 2023)
	THC electrochemical oxidation on MIP-MWCNTs modified SPCE	Plasma	0.17 ng/mL	THC-COOH, THC-OH, norepinephrine, 6-acetylmorphine, stanozolol, ascorbic acid, hydrocodone, buprenorphine, serotonin, dopamine, norbuprenorphine, ethylmorphine, acetaminophen, uric acid, amphetamine, and MTH	(Zhao et al. 2022)
	FcMMA electrochemical-oxidation on MIP NPs modified SPGE	Plasma	16.1 ng/mL	Glucose, C4-HSL, paracetamol, trypsin	(Garcia-Cruz et al. 2020)
	THC electrochemical oxidation on MWCNTs or carbon beads-MIP in micropipette electrode	Buffer	0.18 ng/mL	Caffeine, acetaminophen	(Zhang et al. 2019)
SWV	THC electrochemical oxidation on THC electrodeposited SPCE	Simulated saliva and diluted saliva	1.6 ng/mL	None	(Ortega et al. 2022)
	THC electrochemical oxidation on MWCNTs modified SPCE	Diluted saliva	161.3 ng/mL	Ethanol	(Mishra et al. 2020)
	Competitive binding between THC and BSA-THC with Au NPs functionalized THC-antibody immobilized on Au electrode	Buffer, Urine	7 pg/mL (in buffer)	Morphine, benzoylecgonine	(Eissa et al. 2019)
EIS	THC recognition by THC-antibody immobilized on Au electrode	Saliva	100 pg/mL	pH	(Stevenson et al. 2019)
Current pulse (capacitive)	THC recognition on MIP NPs modified Au electrode	Buffer	N/A	Cannabidivarin, THC-COOH, cannabigerol	(Canfarotta et al. 2018)
FET	THC electrochemical oxidation at the Pt gate electrode	DI water and synthetic saliva buffer	0.32 ng/mL	None	(Majak et al. 2021)
Conductimetric	THC adsorption on semiconducting SWCNTs	Vapor and synthetic breath	0.163 ng (cumulated)	m-xylene, olivetol	(Hwang et al. 2019)

Williams et al. developed a simple method to detect THC at a concentration range of 1–20 μM in 50% diluted artificial saliva (with 0.1 M phosphate-buffered saline [PBS] at pH 11). They used screen-printed carbon electrodes (SPCEs) to electrochemically oxidize THC using DPV (Williams et al. 2023). However, this device lacked sensitivity as the LOD reached 130 nM. Pholsiri et al. developed a copper-phthalocyanine (CuPc)-modified screen-printed graphene electrodes (SPGEs) electrochemical sensor for the simultaneous detection of THC and thiocyanate in diluted saliva samples (with 20:80 ethanol:0.1 M PBS at pH 7) by DPV (Pholsiri et al. 2023). CuPc/SPGE increased the electrical signal of THC oxidation peak current compared with SPGE only owing to the electrocatalytic properties of CuPc for phenolic compound oxidation. The use of TX-100 surfactant improved THC solubility and boosted the signal. The robustness of the device was confirmed by an interference study with a variety of ions/molecules existing in saliva. Under optimal conditions, a linear response was achieved for the THC concentration range of 10–1500 ng/mL with a LOD of 1.37 ng/mL.

Another approach was developed by Kekedy-Nagy et al*.* with an “on–off” electrochemical aptasensor combined with microfluidics cartridge system for the detection of THC in saliva by DPV (Kekedy-Nagy et al. 2023). The assay relied on the competitive binding between THC and a soluble redox indicator methylene blue. The thiol-modified aptamer was covalently attached to gold screen-printed electrodes. Measurements performed in small volume samples (60 μL) showed a LOD of 1 nM in buffer and in the presence of 10% diluted saliva (previously filtered), it increased to 5 nM. The aptasensor was stable for 3 days when stored in dry conditions at 4 °C; however, the reusability dropped from 10 cycles (when freshly prepared) to 5 cycles.

A different technology was developed by Zhao et al*.* with a molecular imprinted polymer (MIP)-based sensor on multi-walled carbon nanotube (MWCNT)-modified SPCE for the detection of THC (by electrochemical oxidation) in plasma by DPV (Zhao et al. 2022). The MWCNT nanostructures were electrodeposited on the SPCE (MWCNTs/SPCE), and the MIP was electropolymerised on the MWCNTs/SPCE surface. Results demonstrated a LOD of 0.37 ng/mL and a linear response in the concentration range of 0–3150 ng/mL in buffer (0.1 M PBS, pH 7). The robustness of the device was confirmed by an interference study with a variety of THC metabolites and other molecules existing in serum. The sensor performances were benchmarked with an ELISA test for the detection of THC in serum with acceptable relative standard deviation (≥ 4.25%) and relative recovery (≥ 99.75%) values.

Continuing with the MIP technology, Garcia-Cruz et al. developed a MIP-NPs modified SPGE including a ferrocene redox probe embedded in the MIP for the detection of THC in plasma by DPV (Garcia-Cruz et al. 2020). MIP-NPs were covalently attached on SPGE using thioalkane linkers. THC was detected with an LOD of 500 nM in a concentration range of 0.1–1000 μM in spiked plasma. No cross-reactivity was observed for cannabidivarin, THC-COOH, and caffeine.

Sensitivity was increased in the work of Zhang et al., who developed MIP particles with carbon cores embedded in a micropipette tube as a working electrode (Zhang et al. 2019). The carbon material was either carbon nanotubes or carbon beads, resulting in a high surface area. The sensor performance for THC detection in 0.4 M potassium chloride solution:methanol (1:1) was evaluated by measuring the electrochemical oxidation peak current of THC using DPV at a concentration range of 10–250 ng/mL. The LOD was found to be 0.32 ± 0.02 ng/mL and 0.18 ± 0.02 ng/mL for MIP-carbon beads and MIP-carbon nanotubes, respectively.

A different measurement method was performed by Canfarotta et al*.* who developed a MIP-NPs capacitive sensor for the detection of THC in buffer (10 mM phosphate, pH = 7.4) (Canfarotta et al. 2018). MIP NPs were assembled by click-chemistry on the Au electrode surface. The capacitive measurements were performed using the current pulse method, which is based on the principle of an electrical double layer for THC concentrations ranging from 1 pM to 10 μM without cross-sensitivity for cannabidivarin, THC-COOH, or cannabigerol.

Ortega et al*.* developed a biochemical-free electrochemical sensor by electrodepositing THC on SPCE (Zensor R&D, Taiwan, Republic of China) for the detection of THC in buffer, simulated saliva, and real saliva by SWV. The modified electrode with THC electrodeposited was expected to better interact with free THC in the solution (Ortega et al. 2022). The saliva effect was handled by subtracting the signals of the interferences with a pristine analog electrode in the same sample. With this approach, the limits of detection were 1.1 ng/mL (in PBS pH = 7.4) and 1.6 ng/mL in simulated and real saliva at the concentration range from 2 to 25 ng/mL. Finally, the technology was benchmarked with a commercial ELISA test (Product No. 120519 from Neogen Corporation) with a confidence level of 95%.

Mishra et al. developed a ring-based sensing platform containing a voltammetric THC sensor and an alcohol amperometric sensor for direct salivary detection in 3 min (Mishra et al. 2020). For THC sensing, 1% of MWCNT was mixed with carbon ink for the SPCE where the electrochemical oxidation of THC was recorded by SWV. THC could be measured in a linear manner in diluted saliva (1:10 saliva:PBS) at a concentration range of 1–4 μM and a LOD of 0.5 μM. While the LOD is rather high, no cross-sensitivity was observed with the detection of alcohol.

Eissa et al*.* developed a multiplexed immunosensor using an array of SPCE for the detection of THC, morphine, and benzoylecgonine. AuNPs were first electrodeposited to increase the specific area and then analyte-antibody was immobilized by self-assembled-monolayer (SAM) and click-chemistry (Eissa et al. 2019). The free analyte in the sample competed with its corresponding BSA-conjugated analyte for the immobilized antibodies on the sensor surface. SWV was performed for the detection, using a 5-mM solution of ferrocyanide/ferricyanide and monitoring the reduction current peak for a simultaneous detection in 20–40 min. The decrease in the SWV peak current was due to the binding of the BSA conjugate, hindering the reduction of the redox probe. A linear response was observed at a concentration range of 10 pg/mL–10 μg/mL with a LOD of 7 pg/mL for THC (0.1 M PBS, pH 7.4). Urine spiked with 10 ng/mL of THC was analyzed with a good recovery rate of 88%.

Similarly, Stevenson et al. developed an impedimetric affinity-based biosensor that utilizes non-faradaic EIS to detect the presence of a BSA-THC hapten in saliva samples (Stevenson et al. 2019). THC antibodies were covalently attached to an Au electrode by SAM and click-chemistry. The sensing response was recorded for a concentration range of 100 pg/mL to 10 μg/mL by spiking saliva with THC. A LOD of 100 pg/mL in varying salivary pHs was achieved, demonstrating stable, dose-dependent biosensing. A binary classification system was employed to predict the presence of THC.

Besides electrochemical sensors, few other isolated technologies have been identified. Majak et al. (Majak et al. 2021) developed unfunctionalized organic electrochemical transistors (OECTs) for the detection of THC in DI water and saliva. The OECTs were fabricated by aerosol jet printing and a Pt wire was used as a gate electrode. The sensing principle was based on the electrochemical oxidation of THC at the gate electrode which induced a drop of the potential applied at the channel/electrolyte interface. The sensor response was recorded at THC concentration between 1 nM to 5 μM with a LOD of 0.1 nM in buffer. Sensors demonstrated less than 3% error indicating good repeatability which was averaged over 15 measurements on several devices.

Hwang et al. developed a conductimetric chemical sensor using semiconductor-enriched single-walled carbon nanotubes (SWCNTs) for the detection of THC in vapor (Hwang et al. 2019). Vapor was generated using a bubbling system for the creation of synthetic breath. The bubbler was filled with water and 0.1%vol of ethanol, 0.1%vol of acetone, and 5%vol of carbon dioxide were added to the gas stream from a calibrated bottle. SWCNTs were immobilized between interdigitated electrodes on a silicon chip by dielectrophoresis forming a thin layer. The silicon chip was incorporated into a hand-held breathalyzer that enclosed an Arduino microcontroller to record the resistance changes of the sensor. The sensing mechanism was based on the strong adsorption of THC on SWCNTs, which remains and disallows the full recovery of the sensor (unlike ethanol, which desorbs in the recovery phase). The use of machine learning algorithms improved the selective detection of THC with better accuracy and the LOD of the cumulated THC was 0.163 ng.

### Commercially available THC testing devices

The need for drug testing has been amplified because of the growing prevalence of illegal drug usage. Hospital laboratories frequently employ immunological methods and automated analyzers to screen illicit drug users. Additionally, there is a rising trend in the adoption of on-site drugs-of-abuse testing devices, not only in laboratories but also in schools, workplaces, and prisons, and for identifying drivers who are under the influence of drugs. Conducting these tests outside of traditional laboratories, often by untrained individuals, poses unique challenges for on-site drug testing. It is crucial that the tests are straightforward and uncomplicated to perform, and the interpretation of results is clear-cut. Above all, tests should not yield inaccurate positive or negative outcomes. Despite the widespread use of on-site drug testing, there is a scarcity of published comparative studies evaluating different test kits. A non-exhaustive list of ELISA tests, with their characteristics, is shown in Table 4.

**Table 4:** ELISA tests commercially available

Product name	Company	Sensing principle	Sensing medium	Cut-off (ng/mL)	Detection time	Reference
RapidSTAT®	MAVAND Solutions GmbH (Germany)	Colorimetric Immunoassay	Saliva	5	6–13 min	(Liut et al. 2022)
DrugWipe®	Securitec (Germany)	Colorimetric Immunoassay	Saliva	10	5 min	(Liut et al. 2022; McCartney et al. 2022; Tang et al. 2018)
DrugTest® 5000	Drägerwerk AG	Colorimetric Immunoassay	Saliva	5, 25	< 9 min	(Dobri et al. 2019; McCartney et al. 2022; Swortwood et al. 2016)
DrugScreen® 5TK and 7TR	IVD-Bio-Innovation (Greece)	Colorimetric Immunoassay	Urine	150	5 min	(Liut et al. 2022)
Randox Evidence® DOA I Plus array (not portable)	Randox Laboratories Ltd. (UK)	Immunoassay	Whole blood, urine, saliva	15	30–60 min	(Efeoglu Ozseker et al. 2023)
OraLine®	Preferred Drug Testing (USA)	Colorimetric Immunoassay	Saliva	4	8–16 h	(Dobri et al. 2019)
OrAlert®	American screening corp (USA)	Colorimetric Immunoassay	Saliva	50	6–12 h	(Dobri et al. 2019)
NarcoCheck®	Kappa City Biotech SAS (France)	Colorimetric Immunoassay	Saliva	10	10–12 min	(Hayden et al. 2022; Thapa et al. 2020)
Fingerprint Drug Screening Cartridges®	Intelligent Fingerprinting Ltd. (UK)	Fluorescent Immunoassay	Sweat (fingerprint)	190 pg/fingerprint	< 10 min	(Hudson et al. 2019)
Alere™ DDS®2	Alere Toxicology (now Abbott, UK)	Immunoassay	Saliva	5 or 25	N/A	(Swortwood et al. 2016)
Ora-Check®	Safecare Biotech (China)	Colorimetric Immunoassay	Saliva	50	5–10 min	(Tang et al. 2018)
THC Ultra Forensic ELISA®	NEOGEN (USA)	Colorimetric immunoassay	Saliva	2.35	120 min	N/A

*UK* United Kingdom, *US* United States

Overall, commercial on-site THC sensing technology is dominated by immunoassay tests due to the robustness of the technology. Saliva is the preferred biofluid because of its non-invasive mode of collection. THC cut-off values are rather varied and range between 2.35 and 190 ng/mL, with analysis time ranging from 5 min to 16 h. A particularly interesting approach is from Intelligent Fingerprint, which collects the sweat of the fingers on a cartridge that is then inserted into a portable reader and results are obtained in 10 min. In addition, Randox Laboratories have developed equipment (Randox Evidence) with a fully automated system able to run 180 samples at once and up to 3960 tests per hour; however, the device is not portable.

**Fig. 5:**
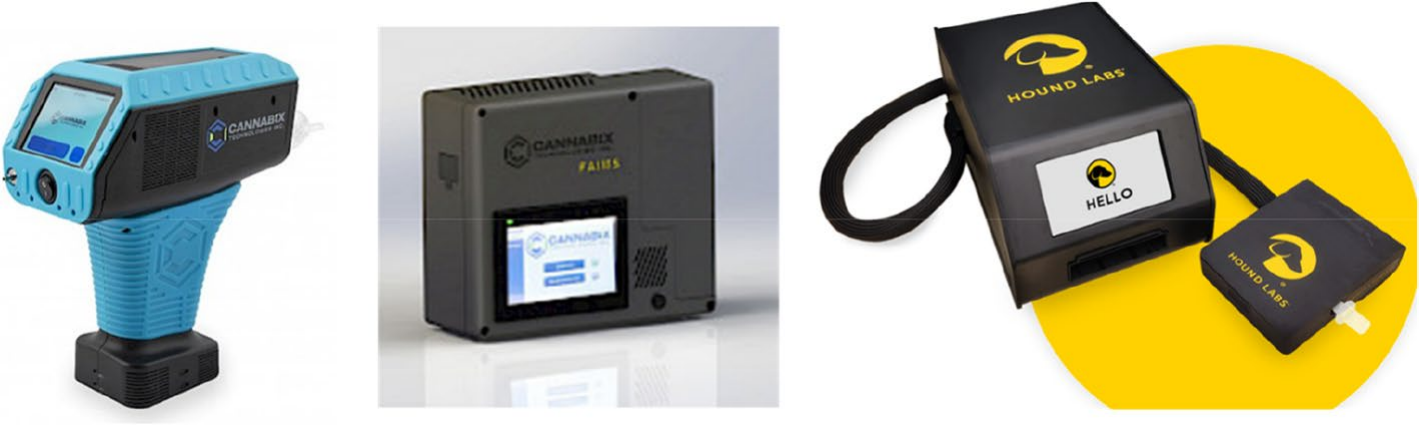
From left to right; Cannabix® THC Breath Analyzer, FAIMS® THC Breathalyzer System, and the Hound®Cannabis Breathalyzer

Recently, several start-up companies have initiated the development of portable breath analyzers for the detection of THC following the consumption of cannabis < 3 h (Fig. 5). Hound Labs is developing the Hound®Cannabis Breathalyzer, the sensing technology which is based on the use of a dye, diazonium salt of rhodamine 123, that reacts with THC and changes its fluorescence. The breath needs to be captured first with a breath capture module, packed in a bed of silica beads, and further eluted with ethanol to strip THC from the module for chemical assay with sensitivity down to a few pg/L.

Cannabix Technologies Inc. is developing two kinds of breath analyzers. The first is based on high-field asymmetric waveform ion mobility spectrometry (FAIMS), acting as a filter which allows a single type of ion of interest (here, THC) at a time to be isolated from background, detected, and potentially quantified. The Cannabix FAIMS® device is used in combination with the breath collection unit. The second device, the THC Breath Analyzer®, uses microfluidic sensors coupled with machine learning algorithms and operates under principles similar to mammalian olfaction systems. Microfluidic-based artificial olfaction technology pairs gas sensors, such as a metal–oxide–semiconductor, with microfluidic channels for the detection of THC in breath samples. The THC Breath Analyzer® collects a single breath from the user and the sensor response is represented by a characteristic pattern or ‘smell-print’ and provides the positive or negative detection of THC in breath. All of these devices are at the advanced prototype stage and not yet commercially available.

There is a clear need for portable systems for rapid, reliable, and quantifiable detection of THC, preferably in saliva or sweat (non-invasive medium and facile to collect). Notably, colorimetric LFIAs are principally utilized owing to their simplicity and ease of use, cost-efficiency, and robustness. An analysis typically involves a few steps, such as adding a sample to the test strip and waiting for the results to appear. This ease of use makes the LFIA accessible to both skilled professionals and untrained individuals. LFIA test kits are generally stable and have a long shelf life when stored properly. This ensures the availability of reliable and ready-to-use tests even in remote or low-resource settings. Furthermore, multiplexing to detect a few analytes is possible on the same strip or with parallel strips. Immunoassays have certain limitations and disadvantages, particularly for quantitative analysis. For instance, all the commercial kits encountered are designed to be semi-quantitative, providing a positive or negative result (based on the cut-off concentration value provided by the supplier), and when positive, an additional analysis is required by conventional method for quantification. Additionally, the cut-off concentrations for some commercial kits are well above the legislated limits in some countries. An interesting trend was identified in the tuning of commercial LFIAs with a complementary optical sensor, allowing quantification of THC in a specific concentration range. The optical signal intensity of the test line was proportional to the THC concentration range.

Electrochemical sensors offer numerous benefits, including the potential for miniaturization, various modifications to enhance sensitivity and selectivity, and cost-effectiveness with rapid responses. These sensors have proven to be sensitive in detecting trace amounts of THC in oral fluids. Nonetheless, there are notable challenges associated with these sensors, such as non-specific interactions and the interference of compounds and substances present in oral fluid. Consequently, the primary issue that the scientific and research community must address is the development of stable and durable electrochemical sensors that exhibit superior selectivity, minimal non-specific interactions, and reduced interference from the matrix before their introduction to the market.

### Uncertainties on THC quantification and open questions

Legal limits for THC in most European nations exclusively pertain to THC levels in blood plasma. Conversely, in Canada and numerous states across the US, there are also established legal limits for THC detected in oral fluid. In contradistinction, urine, sweat, and exhaled breath are considered to have a weaker correlation with plasma concentrations, with probably the smallest deviations in saliva and breath but higher in urine and sweat. It appears that careful correlation studies are still required. Regarding plasma specimens, several legal jurisdictions, encompassing various US states and Canada, have established per se THC limits of either 2 or 5 ng/mL. In contrast, numerous European nations generally maintain a limit of 1 ng/mL, although exceptions include the Czech Republic and the UK with a limit of 2 ng/mL, as well as the Netherlands with a limit of 3 ng/mL (Preuss et al. 2021; Wennberg et al. 2023).

In addition to individual factors, including genetics, diet, and adaptation to cannabis use, the absorption kinetics of cannabinoids and THC depends on the exposure route, with inhalation reaching peak serum concentrations in < 30 min, and ingestion peaking in concentration at approximately 2–4 h (or longer) after consumption. The duration of toxicity secondary to inhalation and ingestion is approximately 2–6 h and 8–12 h, respectively, and the impairment window following inhalation is approximately 3 h (DeGregorio et al. 2021). DeGregorio et al. concluded that as “legalization of medicinal and recreational cannabis continues to expand throughout the US and worldwide, so too does the need for an objective means of determining recent cannabis use and impairment, which cannot be established using currently available breath-based or blood-based testing methods” (DeGregorio et al. 2021). Similarly, another review concluded that some studies show a significant correlation between high THC blood concentrations and car crash risk but this was not to the point of lower THC concentrations (Preuss et al. 2021). Even more confirmatory research appears to be required to investigate quantitative correlations between acute THC use and THC levels in urine and sweat, and how these correlate with the concentrations in the most preferred matrix, blood plasma (DeGregorio et al. 2021; Wurz and DeGregorio 2022). Another open question that is controversially discussed and requires further scientific research remains the degree of impairment that may individually vary in occasional vs. adapted long-term cannabis users or in people with different metabolic phenotypes (Arkell et al. 2019; 2020; Peng et al. 2020; Kebir et al. 2018). Although these open scientific questions will probably require the best available laboratory-based analytical methodology for clarification, we think that there is also a demand for the emerging portable, sensor-based technologies discussed in this review, be it for everyday use in traffic and occupational safety, for large cohort field studies, or home use.

## Conclusions

This review discusses the latest trends in the development of portable technologies and commercial products to detect THC in biofluids. First, THC blood levels ≥ 5 µg/L are known to be associated with a significant increase in crash risk and the legal THC concentration limit in plasma is 1–5 ng/mL in most regulated countries. However, it remains unclear how THC levels correlate with other biofluids.

Conventional analytical methods represent an important segment of the sensing technologies identified because of their accuracy and low LOD (0.1–1 ng/mL). Nevertheless, these methods are notably unsuitable for on-site analysis given their reliance on non-portable laboratory equipment. Additionally, they entail extended result processing periods, ranging from hours to days, and involve intricate methodologies demanding specialized expertise. The detection of cannabinoids, with a primary focus on quantifying THC, has found firm grounding within various analytical separation methods, including high-performance LC and gas chromatography.

The benefits of portable biosensors encompass a resilient setup, mobility, rapid results, and a user-friendly interface. Nonetheless, these benefits are counterbalanced by trade-offs, including reduced sensitivity and precision in comparison to conventional technologies. Two main categories of portable biosensors were identified in this review: optical and electrochemical biosensors. Optical-based biosensors include various techniques such as SERS, ultraviolet–visible-near infrared spectroscopy, colorimetry, and chemiluminescence detection with THC LODs varying from 0.01 to 10 ng/mL. Notably, LFIA appeared to be the most commercially used on-site THC biosensor with cut-off values of 4–190 ng/mL, above the range of regulated THC levels. Additionally, these kits are only semi-quantitative and require additional validation tests.

Extensive exploration has been carried out in the field of electrochemical biosensing as a promising strategy for conducting on-site testing for THC. This is partly attributable to the convenience of converting electrodes into portable, disposable sensors. Conventional immunoassays utilizing pairs of antibodies for THC detection and signal generation face difficulties in adaptation because of THC’s relatively small molecular size. These circumstances enhance the appeal of electrochemical sensors because such sensors typically rely on inherent THC redox reactions, indirect chemical processes, or impedance spectroscopy to generate signals. Nonetheless, these techniques are also susceptible to variations in environmental conditions, such as electrolyte composition, the presence of metabolites, and pH levels. These factors can interfere with electrical measurements, potentially resulting in a diminished signal-to-noise ratio that necessitates external signal amplification.

This review described the recent cutting-edge portable technologies for the detection of THC in biofluids. It also indicated promising potential for the rapid on-site determination of THC in biofluids. The principal goals for optimizing portable biosensors involve their miniaturization, while still maintaining ample sensitivity and selectivity, alongside cost-effectiveness and fast response times. Nevertheless, the primary challenges in the advancement of portable biosensors encompass non-specific interactions and the potential interference of substances within the sample matrix.

### Supplementary Information

**Additional file 1.** Search methodology.

**Additional file 2.** PRISMA flow diagram.

## Data Availability

Not applicable.

## Abbreviations

AuNPab: AuNP-THC antibody
BSA: Bovine serum albumin
CBD: Cannabidiol
CuPc: Copper-phthalocyanine
DPV: Differential pulsed voltammetry
ELISA: Enzyme-linked immunosorbent assay
HRP: Horseradish peroxidase
IgG: Immunoglobulin G
LC: Liquid chromatography
LFIA: Lateral flow immunoassay
LOD: Limit of detection
MIP: Molecular imprinted polymer
MS: Mass spectroscopy
MWCNT: Multi-walled carbon nanotube
NP: Nanoparticle
PBS: Phosphate-buffered saline
SA: Streptavidin
SAM: Self-assembled monolayer
SERS: Surface-enhanced Raman spectroscopy
SPCE: Screen-printed carbon electrode
SPGE: Screen-printed graphene electrode
SWCNT: Single-walled carbon nanotube
SWV: Square wave voltammetry
THC: Δ9-Tetrahydrocannabinol
UCNP: Upconverting nanoparticle
UK: United Kingdom
US: United States
