# Supplementary material for: Recent advances in the development of portable technologies and commercial products to detect Δ9-tetrahydrocannabinol in biofluids: a systematic review
Source: J Cannabis Res. 2024 Feb 27;6:9. doi: 10.1186/s42238-024-00216-0 (PMC10898188; doi:10.1186/s42238-024-00216-0)
Supplement: Supplementary file 2 — Additional file 2. PRISMA flow diagram. [file 42238_2024_216_MOESM2_ESM.docx]

**Identification of studies via databases and registers**

Records removed *before screening* (n = 8709):

-Not a report of detection/quantification

-Detection not in biofluid

-Synthetic cannabinoids

-Wrong publication type (report/letter with no data)

-Post-mortem analysis

-Other body fluids (breast milk, semen)

-Duplicate records

-Language issue

Records identified from data bases searching (n = 8893); Pubmed (n = 3892), Scopus (n = 2441), Google Scholar (n = 2560)

**Identification**

**Screening**

Full-text papers assessed for eligibility (n = 89);

-Chromatography (n = 22)

-Optical sensors (n = 12)

-Electrochemical sensors (n = 13)

-Commercial sensors (n = 7)

Records screened (n = 184)

Records excluded (n = 95):

-Results not clearly presented and/or beyond the scope of the review

Full-text papers excluded

(n = 35);

- chromatographic techniques fall beyond the coverage of this review so we have chosen only articles from 2020 onwards

Studies included in the review

(n = 53)

**Included**

*From:*  Page MJ, McKenzie JE, Bossuyt PM, Boutron I, Hoffmann TC, Mulrow CD, et al. The PRISMA 2020 statement: an updated guideline for reporting systematic reviews. BMJ 2021;372:n71. doi: 10.1136/bmj.n71

For more information, visit: <http://www.prisma-statement.org/>
